# Healthcare professionals’ attitudes towards social prescribing in specialist children’s weight management services

**DOI:** 10.1186/s12875-025-02753-w

**Published:** 2025-02-26

**Authors:** Megan Garside, Catherine Homer, Christopher Dayson, Lorna Dowrick, Katie Pickering, Neil Wright

**Affiliations:** 1 Advanced Wellbeing Research Centre, Sheffield Hallam University, Sheffield Hallam University, Olympic Legacy Park, 2 Old Hall Road, Sheffield, S9 3TU UK; 2 Sheffield Children’s Hospital, Clarkson St, Broomhall, Sheffield, S10 2TH UK

**Keywords:** Children, Young people, Social prescribing, Weight management, Obesity

## Abstract

**Background:**

Addressing increasing rates of childhood obesity is a global priority. High numbers of children and young people are living with obesity and experience significant physical and mental health impacts. Social prescribing research has shown it can help improve young people’s physical and mental health, meaning it may be a helpful way to provide additional, personalised support to young people who are living with obesity, and to help to address the health inequalities experienced by this group. This study aimed to provide an overview of the current understanding and use of social prescribing from healthcare professionals working in specialist weight management services in England, and to identify perceived benefits and barriers to future implementation of social prescribing in these services.

**Methods:**

A national survey was distributed online between April and July 2023 to healthcare professionals working within specialist weight management clinics across England to gather information regarding their current use and understanding of social prescribing.

**Results:**

Thirty-eight completed surveys were analysed, with good representation from services across England. Staff felt they had an understanding of what social prescribing is and were willing to use it. Anticipated benefits included improvements to wellbeing and providing opportunities for physical activity and family support. Reported barriers included limited capacity from staff and a need for more training around how to identify appropriate community-based services to link with.

**Conclusion:**

Healthcare professionals working in children’s weight management services felt social prescribing could be beneficial for the families they worked with. However, to support implementation in their services, there is a need for further resource, such as staff time and training, to help develop relationships between clinical services and community-based services.

## Background

### Obesity

Childhood obesity is a major public health challenge worldwide [1, 2] with estimates that almost 20% of 5–19 year olds were living with overweight or obesity in 2022 [2]. In England, data from the National Child Measurement Programme 2023/24 reports 9.6% of children aged 4–5 years and 22.1% of children aged 10–11 years are living with obesity [3]. This data also suggests that this group experiences significant health inequalities, with children living in the most deprived areas of England being more likely to be living with obesity with a prevalence rate of at least double that experienced by children living in less deprived areas [3].

Excess weight in childhood has been linked to a range of significant physical health impacts such as cardiovascular disease, Type 2 diabetes, high cholesterol, and musculoskeletal issues [4, 5] as well as mental health challenges, such as low self-esteem and low self-confidence [6]. Many of these physical and mental health impacts can continue into adulthood [7] with significant cost to health services [8]. Obesity is therefore a key health priority to address, and in England, there are targets to reduce rates of obesity included in the National Health Service (NHS) Long Term Plan [9].

Models of weight management support across England have been organised into four Tiers depending on the type of support offered [10, 11]. NICE guidance [12] recommends support approaches should be tailored to the needs of families, by developing services with input from local community engagement and multidisciplinary healthcare teams.

Tier 1 services include universal public health measures, such as national healthy lifestyle campaigns and advice from professionals like GPs and school nurses. Referrals can be made to Tier 2 services if a family needs further support, and these services include community weight management programmes generally consisting of time-limited, group courses focusing on lifestyle changes. Tier 3 services are more intensive weight management programmes delivered by a multi-disciplinary team of healthcare professionals (including consultants, nurses, dieticians, nutritionists, physiotherapists, psychologists) and a focus on underlying issues such as mental health difficulties. They are recommended when additional support is needed after accessing Tier 2 services, after relapse, or if an individual experiences significant co-morbidities [13]. Tier 4 services include more intensive surgical and non-surgical medical treatments.

Despite high levels of need, nationally, there are no formal standards and limited availability of services for children and young people at Tier 3 level. In response, in 2022, NHS England commissioned twenty-one Complications from Excess Weight (CEW) clinics to provide support to CYP living with severe obesity at Tier 3 level. This has been increased to up to forty-three clinics from 2024. These CEW clinics [14] support children and young people aged under 18 years who have or are at risk of developing significant complications related to their weight, and who need further support after accessing Tier 2 services. The CEW clinics are built on a biopsychosocial and holistic approach, aiming to consider underlying factors which may contribute to the development of severe obesity including mental health, physical health, and social needs. The delivery models at each of the clinics vary based on local needs but each aim to create holistic, person-centred packages of care for families. There is a recognised need for this care to be personalised, acceptable and accessible to all families, and many clinics employ support workers who can provide additional home visits and community-based support to families.

Whilst a multi-disciplinary team of healthcare professionals is essential to providing care for families at Tier 3 level, to improve long term outcomes there is also a need for families to be able to access ongoing and sustained support outside of the clinic environment, such as at home or in their local communities. For example, for accessing low level mental health support and opportunities for physical activity, it is possible for community-based services to provide this in non-stigmatising, accessible locations, with reduced waiting lists, and long term support which can continue after clinic-based treatment has ended [15, 16]. This has the potential to be achieved through social prescribing.

### Social prescribing

Social prescribing involves healthcare professionals referring patients to community-based services to support them with their wellbeing [16]. These services can include (but are not limited to) activities such as sports, crafts, volunteering, or gaming groups. This process often works through referral to a ‘link worker’ who provides holistic support and a patient-centred, flexible approach to support an individual to choose and access a community service [17].

Whilst social prescribing was originally implemented in healthcare services in the United Kingdom (UK), it has since become a global concept, with social prescribing initiatives reported in over 30 countries [18, 19]. There is an International Social Prescribing Collaborative, with ambitions to share learning across countries [19].

In the UK there has been increasing interest and investment in social prescribing over recent years, with commitments to embed social prescribing services and link workers into Primary Care Networks outlined in the NHS Long Term Plan [9]. In adult populations, research has found positive results from social prescribing in terms of improvements to wellbeing, reductions in A&E attendances and reduced demand for GP services [20, 21]. However, there have been calls for more robust research in this area to build a more definitive evidence base [22], with current studies often showing wide variation in delivery models and poor evaluation design, which limits conclusions [23].

For social prescribing with children and young people, the evidence base has increased over recent years [24]. A recent systematic review highlighted preliminary evidence to suggest improvements to wellbeing, and a favourable return on investment [24] although these studies only included those over 15 years old. These findings align with reports which demonstrate improvements for young people’s mental health, reductions in social isolation, and wider benefits such as reduced health inequalities [25, 26]. Social prescribing programmes can also provide regular and sustainable opportunities for physical activity, in an accessible and non-stigmatising environment [15].

Families accessing CEW clinics often experience complex social challenges and are living in more deprived areas of the country [27]. In particular, young people living with severe obesity commonly experience low mood and low self-esteem [27]. Based on the understanding that social prescribing is a holistic, person-centred form of support, which can support young people with their mental health, reduce isolation, provide accessible opportunities for physical activity and develop confidence [25, 26], social prescribing may be a helpful way to provide additional ongoing, accessible support to young people alongside CEW clinic treatment.

Currently, social prescribing programmes are not formally included in the CEW clinic pathway for children and young people. All Primary Care Networks in England offer an all age link worker resource but youth social prescribing provision varies significantly across local areas. This current paper details a national survey for professionals working in CEW services. The aim was to understand if there were any examples of social prescribing programmes across clinics, as well as to gather professionals’ understanding of social prescribing and potential barriers and needs for implementation.

## Method

### Design

This study used a cross-sectional, quantitative survey design, with data collected from professionals working as part of Complications of Excess Weight (CEW) clinics across England. Ethical approval was obtained in April 2023 from Sheffield Hallam University Ethics Committee. The survey was designed and delivered online using Qualtrics [28].

### Survey questions

Questions were bespoke and informed by similar surveys of social prescribing [29] and through initial engagement with CEW clinic staff. Questions included closed text box responses, and options to respond with free text. The survey collected demographic details of respondents including their job role, type of organisation and which CEW clinic they were part of.

Questions explored *current understanding of social prescribing*, by asking professionals to define what they thought social prescribing was, if they had heard of the term before, and how much they knew about it.

Questions then focused on their *current use of social prescribing*. A definition of social prescribing was provided based on online resources [30, 31]: *For this project*, *we describe social prescribing as healthcare professionals referring individuals to access local community-based groups (such as sports clubs*, *arts and crafts groups*, *nature-based activities) which can support them with their health and wellbeing. This can be done in different ways*, *including through direct referral from a healthcare professional to a community-based group*, *or through referral to a ‘link worker’ who works with an individual to support them to choose and access a local community-based group.* Questions asked if they use social prescribing currently in their role or not based on this definition. If yes, they were asked how many children and young people they referred through social prescribing and for examples of the types of services they referred to. If not, they were asked for reasons why they do not currently use it.

Questions then asked about the *anticipated benefits of social prescribing.* All respondents, regardless of whether they currently used social prescribing or not, were asked if they thought social prescribing could be helpful for the children and young people they worked with, and what aspects would be most beneficial. Finally, questions focused on *potential barriers to using social prescribing* in their service. Respondents were asked if they would be likely to use social prescribing in future, and what barriers they may face to implementing it, as well as if they were aware of local community-based services that they could work with.

### Procedure

The online survey link was circulated to lead clinicians at each of the CEW clinics nationally, and they were asked to share this with their local CEW teams and networks. The survey was initially distributed via email by a consultant working within the CEW clinics and a researcher, with a reminder sent two weeks later. A final reminder was sent a month later, two weeks before the survey closed. The initial email distribution list contained approximately 130 contacts. The survey was also shared with contacts at NHS England CEW delivery board meetings and through networks of dietetics and psychologists working at CEW clinics. Respondents were staff working with children and young people (aged 2–18 years old) living with obesity, as part of a CEW clinic.

The first page of the survey provided information about the study, including its scope and aims, and contact details for the researcher. Participants were informed that they could contact the researcher with any questions they had about the research. An information sheet was available to read and download, and consent was required to be provided before the survey could be accessed. The survey was expected to take no more than 10 min to complete. The survey was live for four months, between April and July 2023.

Participants remained anonymous and were not required to provide their name or any contact details. Participants were asked for their job role and which CEW clinic in England they worked for, however these details were to check distribution of responses nationally and were not linked to later responses. The majority of questions asked for closed, tick-box responses but with options for open text responses included. Participants were reminded to not disclose any personal information about themselves or their service.

### Analysis

Survey responses were downloaded into an Excel file. Descriptive analysis, including frequency, counts and percentages [32], was used to summarise responses to quantitative questions, and graphs were created to visually present responses. Descriptive summaries are provided for open text questions, with consistent themes identified across responses. Due to the small sample size, and nature of the questions, statistical analysis was not appropriate.

## Results

### Respondents

The survey received 42 responses in total. Of this number, 38 completed the full survey, three started the survey but did not answer any questions and one started the survey, responded with which clinic they were part of and then did not answer any further questions. Of the 38 who completed the full survey, the survey link was open for a minimum recorded time of 2 min, and the maximum was recorded as 64 h. The median time was 5 min.

There were responses from over 75% of CEW clinics nationally (16 out of 21 clinics) and an additional respondent from NHS England. Most of the clinics (*n* = 14) had 1–3 respondents complete the survey, apart from two clinics which had seven and eight respondents completing. The majority of respondents reported their role to be NHS, apart from one who worked as part of a charity.

27 professionals reported their job role. Respondents included consultants (*n* = 7), dieticians (*n* = 5), psychologists (*n* = 4), family support workers or youth workers (*n* = 4), paediatricians (*n* = 1), nurses (*n* = 1), clinical fellows (*n* = 1), physiotherapists (*n* = 1) and project managers and staff from Children and Young People’s services (*n* = 3).

### Current understanding of social prescribing

30 professionals provided a text response to ‘what do you consider social prescribing to be’? A high proportion of respondents mentioned social prescribing as being a ‘non-medical’ or ‘non-drug intervention’, specifically involving local community-based services (*n* = 21).

Respondents often mentioned the role of the healthcare professional in providing this referral or non-medical prescription (*n* = 12). Others mentioned signposting or providing links to families (*n* = 9), and some respondents mentioned active support for families to access services or directly working with the young person to identify what their interests are (*n* = 2).

There was a general understanding that outcomes of interest for social prescribing are wider and more holistic than just physical health with specific references to mental health, wellbeing, isolation, lifestyle changes and supporting a ‘full’ or ‘fulfilling’ life (*n* = 13).

95% of respondents had previously heard of the term ‘social prescribing’. Over three quarters of respondents (76%) felt they knew ‘a bit about it’ or that they ‘didn’t really know much about it’ (24%). None of the respondents felt that they knew ‘a lot’ about it and none reported that they ‘didn’t know anything about it’.

### Current use of social prescribing

The survey then provided a definition of social prescribing, informed by existing online resources [30, 31] as noted in the *Survey Questions* section above:

Based on this definition, just over half of respondents (55%) felt they used social prescribing in their job role. 45% of respondents did not feel they were using social prescribing.

#### Referrals and services used

For those that did use social prescribing in their role (*n* = 21), 90% referred less than 10 children and young people on average in a month. 10% (*n* = 2) referred between 10 and 20. No respondents were referring more than 20 young people a month to social prescribing. In practice, the number of young people each CEW clinic is commissioned to see varies. When the CEW clinics were first developed, they were each commissioned to see varying numbers of young people, for example this could be between 20 and 200 young people per year depending on the clinic.

18 professionals provided examples of the types of activities they referred young people to. The majority (*n* = 17) of these mentioned some form of physical activity including community sports clubs, leisure centres, community football foundations and local gyms. Seven professionals referred to youth groups, which provided sessions specifically for children and young people. Two professionals included art and craft sessions as part of the services they referred young people to. Two professionals mentioned cooking groups or diet education and three professionals reported therapies, mental health support or sessions specifically for those with additional needs.

#### Reasons for not using social prescribing

**Fig. 1:**
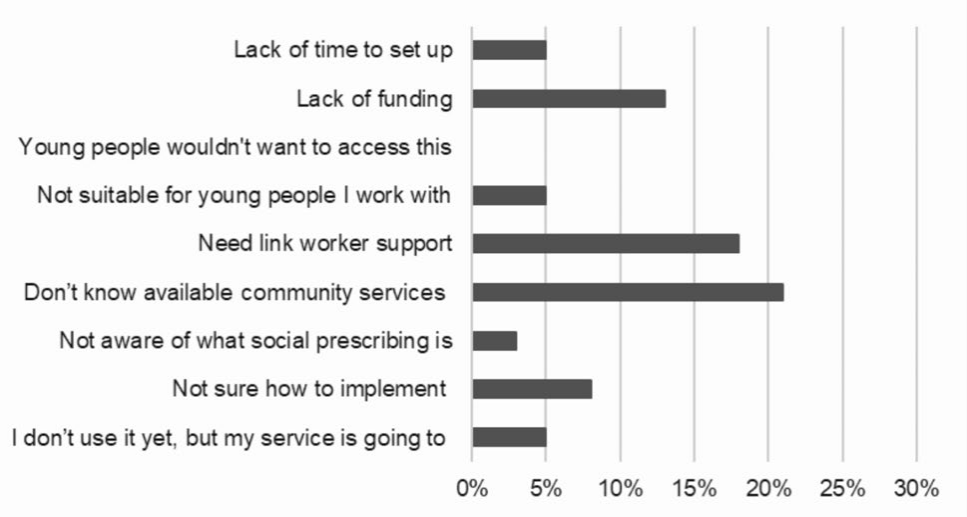
Why do you not use social prescribing currently in your role?

For those professionals who stated they did not currently use social prescribing within their role (*n* = 17), they were asked for reasons why not, selecting as many options as applied (Fig. 1). The main reasons were due to not knowing the available community-based services to refer to (21%), the need for a link worker role to support referrals (18%) and a lack of funding (13%). No professionals felt that the young people they worked with would not want to access this.

Nine professionals provided additional text responses for this question. Some professionals (*n* = 5) reported they did not always have a direct role working with children and young people, and felt social prescribing was the role of other colleagues in their team. Other professionals (*n* = 3) raised an issue of the lack of available services specifically for children and young people, and felt social prescribing services were currently aimed at adults. Another professional (*n* = 1) identified a community-based programme they used in their service, which they did not feel fit the definition of social prescribing as it was specifically designed for their patients.

### Anticipated benefits of social prescribing

All respondents, regardless of if they did or did not currently use social prescribing in their role, were asked if they thought social prescribing could be helpful for children and young people. Most (82%) professionals answered ‘yes’. 8% of professionals responded as ‘not sure’ and 8% responded as ‘sometimes’. No professionals responded as ‘no’.

**Fig. 2:**
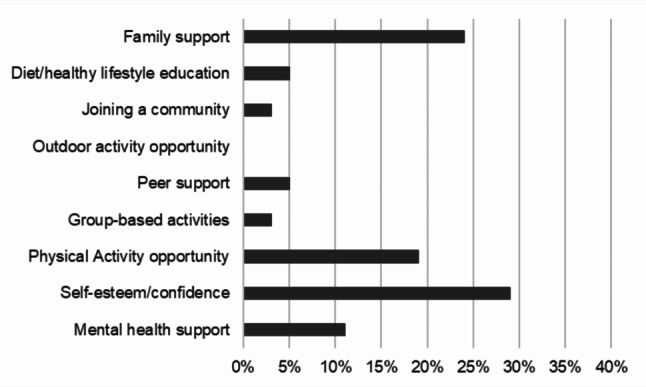
What do you think would be most beneficial for the young people and families you work with, as part of a social prescribing offering?

When asked to consider what would be most beneficial for the young people and families they worked with (by choosing just one option), professionals most commonly selected ‘improving self-esteem and confidence’ (29%), ‘family support – involving children and parents/carers together’ (24%) and ‘opportunities for physical activity’ (18%). See Fig. 2.

The majority of professionals responded that they would be either ‘very likely’ or ‘fairly likely’ to use social prescribing in their role in the future (87%). 8% of professionals responded as ‘not very likely’ and 5% did not know. No professionals felt they were ‘not at all likely’ to use social prescribing in the future.

### Barriers to implementation in services

**Fig. 3:**
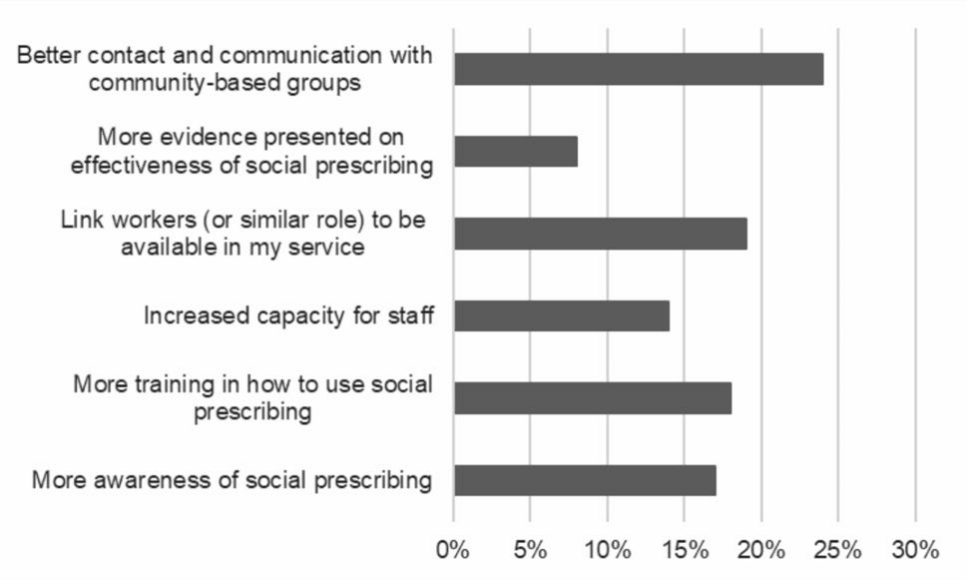
What support would be needed to set up a social prescribing offering in your service?

Professionals were asked what support they might need to set up social prescribing in their service (Fig. 3).

Almost a quarter (24%) of respondents felt they needed better contact and communication with community-based groups. 19% also identified a need for a link worker or similar role to support with referrals and 18% wanted more training on how to use social prescribing.

When asked if they knew which community-based services they could refer young people to in their local area, responses were equally split between ‘yes’ (29%), ‘no’ (26%) and ‘not sure’ (24%). Eight professionals provided additional comments, most commonly reporting they felt they knew some community-based services but needed to know more and identifying challenges around having time and capacity to find these, particularly when their service provided care across large areas (n = 6).

Final additional comments to the survey as a whole were provided by seven professionals. These generally highlighted issues around knowing what community-based services are available and identifying a lack of services specifically for children and young people (*n* = 4).

## Discussion

Social prescribing is gaining recognition and becoming more commonly implemented globally [19] and within the UK, increasingly for children and young people [26]. Despite this, social prescribing is not consistently integrated across all clinical services and has not yet been explored in specialist services such as children’s weight management. This survey provides an overview of the current understanding and use of social prescribing in specialist weight management clinics for children and young people (specifically ‘Complications from Excess Weight’ CEW clinics) across England, as well as perceived benefits and barriers to future implementation.

The increase in use of social prescribing across UK services may be reflected in the survey results, with responses showing that the majority of professionals had heard of social prescribing and understood what it involved. Descriptive responses consistently mentioned ‘non-medical’ support and outcomes relating to meeting wider wellbeing and lifestyle needs of families. This aligns with the wider evidence base, which shows an increase in studies of social prescribing in recent years with focus on wellbeing outcomes such as mental health support and reduced isolation [24].

Professionals’ descriptions of social prescribing generally focused on a direct referral method, with professionals referring to community-based services, rather than referring on to a ‘link worker’ or similar role. Only one respondent mentioned referral to a local service which then connects families to community groups, more similar to a link worker model of social prescribing. This may reflect that whilst primary care networks now offer some level of access to a social prescribing link worker [31], access may still be limited specifically for children and young people. This means that professionals are often referring direct to community-based services they know, with more of a ‘signposting’ approach, rather than to a dedicated link worker who can support this process. Social prescribing can still work through a direct approach, with professionals connecting families to services they know and trust. However, link workers often have a wider knowledge of local services that are available, meaning they may be able to offer the young person more choice over which service they access, or help find a service that is more tailored to the young person’s needs and interests. In addition, link workers may have more capacity to support the young person to access this service, either through increasing their confidence to attend a new group or by supporting with practical aspects, such as how to travel to the group.

Although social prescribing has not yet been formally implemented in the UK to support young people living with severe obesity, there was recognition from professionals that this would be beneficial. Over 80% of professionals thought social prescribing would be helpful for the young people they worked with, and that they would be likely to use it in future. The main identified areas of benefit focused around developing self-esteem and confidence and providing opportunities for physical activity and family support, which involved working with young people and parents/carers together. Similarly, the current evidence for social prescribing with children and young people suggests improvements for confidence, self-esteem, and reduced isolation [24, 25]. In terms of weight management, community-based treatments and family approaches also show high levels of promise [33].

The potential benefits of social prescribing, particularly developing confidence and providing opportunities for physical activity, were also reflected in the examples professionals provided of services they refer families to. Just over half of respondents felt they were using social prescribing in their role, mainly through direct referrals to local community groups or leisure centres and gyms which provided opportunities for physical activity. In a recent social prescribing study for young people accessing mental health services, group physical activity was shown to help facilitate development of trusting relationships and also facilitate improvements to mental wellbeing [15, 34].

The main barriers professionals faced in using social prescribing in their role were related to not knowing the available community-based services to collaborate with and there was a reported need for an additional role in their service to support with this, such as a link worker. However, not knowing the available local services may not be due to a lack of time and capacity but may be due to a lack of suitable community-based services that are ready to be part of a social prescribing offer. Professionals felt there was a lack of services for younger children, with many being adult-focused or targeted at those over 18 years. This is reflected in the literature, with the most recent systematic review of social prescribing for children and young people finding included studies commonly included those over 15 years old [24]. There may therefore be a gap in provision of suitable community-based services, limiting the potential for collaboration between healthcare providers and community organisations through social prescribing. This aligns with reports which call for increased investment into youth services to support the delivery of social prescribing [25, 26].

Free text comments throughout the survey emphasised the workload involved in developing an understanding of what community-based services exist and to develop connections with them. This is an important process, as healthcare staff need to have trust in the services that they are referring service users to, and community-based services need to trust and have good working relationships with the clinical services they take referrals from to support collaborative working [35]. Mapping work has been undertaken in one CEW clinic locality to identify local community-based services that are suitable for this population. Findings from this work are published elsewhere [36] and show the time and resource this mapping work required, as well as the limited availability of community-based services that meet the specific needs of this population. In practice, it may be that this work to map and develop links with community-based services could be undertaken by link workers embedded into the CEW service.

In this current survey, professionals highlighted that this mapping work was often the role of a family support worker, youth worker or social worker, although not all CEW clinics will have this role included in their multidisciplinary team. It is important that training is provided to support social prescribing with 18% of professionals who completed the survey stating training was needed to support implementation. Current training for link workers who deliver social prescribing can be variable across areas but often includes e-learning modules [37]. This e-learning is all-age focused and therefore is unlikely to include sufficient detail to reflect the complexity of working with young people, or with those with specific additional needs, such as for those living with obesity. It is currently targeted at NHS staff, meaning this training is less accessible for those working in community-based services.

### Recommendations

Findings from the survey have been able to inform recommendations for policy and practice for developing a social prescribing offer within weight management services for children and young people. The challenge highlighted by staff in identifying appropriate local community-based services may reflect a lack of available services that meet the needs of young people living with obesity, but it may also be that more support for community services is required, especially if these services are expected to support young people with complex needs. Support for community services, such as increased staff capacity, funding, training and supervision should therefore be considered. Additionally, embedding a youth specific link worker into the clinical multidisciplinary team may increase capacity to develop connections with local community services and provide support to families to access these.

CEW staff reported that social prescribing could support families with developing confidence, providing opportunities for physical activity and family support. Therefore, a social prescribing offer within weight management services should focus on addressing wider, holistic goals, such as mental health and self-esteem rather than focusing solely on physical health outcomes or weight change. This aligns with NICE guidelines which recommend involvement in physical activity, not only to lose weight but also because of the wider benefits this can provide [12]. Developing guidance and training for clinical staff around what social prescribing is, what benefits it can offer, and which families it works best for can help to develop understanding and trust in the service, and support implementation. This has been noted by other social prescribing studies in hospital settings [38].

### Strengths and limitations

The current survey was limited by a small sample size, with 38 full completions of the survey. Despite this, there was good distribution across sites nationally, as well as across roles within the CEW clinics. The aims of the survey were able to be achieved, by providing a current overview of CEW professionals’ understanding of and attitudes to social prescribing in their service.

This current study focused on children’s weight management services within England, meaning findings may not be fully generalisable to other countries, especially given the complexities involved in childhood obesity and variations in healthcare services. However, the findings from this study provide insights into the application of social prescribing to this population and an understanding of potential barriers and needs to support implementation. As childhood obesity is increasingly a global issue [1], alongside the increasing international interest in social prescribing [19], this study provides insights into potential application of social prescribing for this population, which will be of use to healthcare professionals and researchers working within this field across different countries.

## Conclusion

Social prescribing is gaining recognition globally as a way to offer individuals additional, holistic support with their health and wellbeing. Social prescribing could be beneficial for supporting young people living with obesity and their families, particularly in terms of improving wellbeing, providing opportunities for physical activity, and providing family support. However, to allow for implementation into services, there is a need for resource to support this. This support is needed both in terms of investment into community-based services specifically for children and young people, and staff time and training for both clinical and community-based services to support collaboration. Embedding and connecting youth link workers into specialist healthcare services could be explored as a way to support implementation of social prescribing for young people living with obesity.

## Data Availability

The datasets used and/or analysed during the current study are available from the corresponding author on reasonable request.
